# The Emotional Experiences of Healthcare Professionals Working in Amyotrophic Lateral Sclerosis: A Systematic Review

**DOI:** 10.1002/mus.70309

**Published:** 2026-06-18

**Authors:** Young Chan, Sarah Creer, Jess Mere, Alys Wyn Griffiths, Charlotte Wright, Esther V. Hobson, Christopher J. McDermott, Emily J. Mayberry

**Affiliations:** ^1^ Sheffield Institute for Translational Neuroscience, Division of Neuroscience, School of Medicine and Population Health University of Sheffield Sheffield UK; ^2^ Clinical and Applied Psychology Unit University of Sheffield Sheffield UK; ^3^ Sheffield Teaching Hospitals NHS Foundation Trust Sheffield UK

**Keywords:** amyotrophic lateral sclerosis, emotional experiences, healthcare professionals, motor neuron disease, qualitative evidence synthesis

## Abstract

Healthcare professionals (HCPs) working with people living with amyotrophic lateral sclerosis (ALS) are often exposed to emotive circumstances including end of life care, trauma, loss, and death. Existing reviews have explored the emotional experiences of people living with ALS and their carers but have largely ignored healthcare staff and the impact of this work on them. This systematic review of qualitative research aims to explore the emotional experiences of and impact on HCPs working with people living with ALS using thematic synthesis (PROSPERO reference: CRD42025631749). Electronic databases were searched for journal articles and gray literature (Medline, Scopus, PsycINFO, Google Scholar, King's Fund Library Database, ProQuest Dissertations, Theses Global) for qualitative or mixed‐methods studies exploring the emotional impact on HCPs working in ALS. Twelve studies were included, critically appraised, and analyzed. Four themes were identified. The *emotional intensity due to the nature of ALS* created challenges for HCPs, while they were also faced with *absorbing the emotions of others.* HCPs learned to *balance their emotional involvement*, and HCPs also described *positive experiences and coping mechanisms.* HCPs working in ALS experience multi‐faceted emotional challenges, and they do describe positive emotional experiences within their roles. However, HCPs describe having few coping mechanisms and limited formal support systems in place to process the intense emotions or to guide their emotional involvement. The lack of support for staff may ultimately negatively affect patient care. There is an unmet demand for debriefing, supervision, and further training on how to deal with intense, distressing emotions.

**Abbreviations**
ACT: acceptance and commitment therapy
ALS: amyotrophic lateral sclerosis
CASP: Critical Appraisal Skills Programme
ENTREQ: enhancing transparency in reporting the synthesis of qualitative research
HCP: healthcare professional
MeSH: Medical Subject Headings
MND: motor neuron disease
NIV: noninvasive ventilation
PRISMA: Preferred Reporting Items for Systematic reviews and Meta‐Analyses

## 1 Introduction

Amyotrophic lateral sclerosis (ALS) is the predominant subtype of a spectrum of rapidly progressive, terminal neurodegenerative disorders affecting both upper and lower motor neurons [1, 2, 3]. Many people with ALS experience cognitive and/or behavioral changes, and people with ALS and their family members often experience high levels of emotional distress and significantly decreased quality of life [4, 5, 6, 7]. Given the lack of cure, the majority of ALS care consists of supportive measures managed within a coordinated multi‐disciplinary team setting, and this is associated with improved clinical outcomes [8, 9]. This comprises a complex network of healthcare professionals (HCPs), including neurologists, specialist nurses, physiotherapists, occupational therapists, dieticians, speech and language therapists, and specialists from other adjacent disciplines, such as palliative care.

The lived experiences and psychosocial needs of people living with and affected by ALS are increasingly recognized [10, 11, 12, 13, 14], with recent development and evaluation of interventions to manage these [15, 16, 17]. However, the emotional impacts on HCPs working within ALS are often overlooked [18]. A recent scoping review explored HCPs' experiences working within ALS and highlighted the emotional impact as a recurrent theme in the existing literature [19]. However, the emotional impact was not the focus of the previous scoping review, and the review suggested further work is needed to identify the psychosocial and emotional needs of HCPs working in ALS. Therefore, this systematic review aims to develop a deeper understanding of the emotional experiences of HCPs working in ALS, and to describe the impacts of these emotional experiences. By also exploring coping mechanisms, this review will help to inform future staff support.

## 2 Methods

This systematic review was registered on the international prospective register PROSPERO (reference: CRD42025631749) and the enhancing transparency in reporting the synthesis of qualitative research (ENTREQ) statement [20] and the Preferred Reporting Items for Systematic reviews and Meta‐Analyses guidelines and checklist [21] were followed throughout.

### 2.1 Search Strategy

The search strategy, search terms, and databases were identified by the research team in consultation with a librarian. A combination of MeSH terms and free text search terms for each construct were inputted into Medline, Scopus, and PsycINFO (see Supporting Information 1 for full search strategy and terms) in December 2024. Gray literature was also searched (Google Scholar, King's Fund Library Database, ProQuest Dissertations and Thesis) in December 2024. Searches were updated in January 2026. Qualitative and mixed‐methods study designs were included. Review articles were excluded. No limits on year of publication were applied. Search results were limited to publications written in English.

### 2.2 Data Screening

Publications identified via cross‐database searches were extracted. Duplicated results were removed. Titles and abstracts were screened against the eligibility criteria by Y.C. (see Supporting Information 2). Remaining papers were subjected to full‐text review, conducted independently by Y.C. and S.C. Where disagreements arose, Y.C., S.C., and E.J.M. made the final decision through discussion. Forward and backward reference searches of included papers were performed. Quality was assessed using the Critical Appraisal Skills Programme (CASP) qualitative research checklist by both YC and SC [22].

### 2.3 Data Extraction

YC extracted authors, year of publication, country of publication, study design (recruitment strategy, methods of data collection and analysis), number of HCP participants, available characteristics such as age and gender of the participants, experience of providing care to people living with ALS, and key themes from each included report.

### 2.4 Data Analysis

Data were analyzed utilizing Thomas and Harden's thematic analysis approach to meta‐synthesis of qualitative research [23]. This involved extracting findings, including descriptions, interpretations, and verbatim quotations. Only data relating to HCPs' emotional experiences were extracted. A minimum of one quotation related to an emotional experience was necessary from the studies to be included. Data were subject to line‐by‐line coding of participant quotations and researchers' descriptions and interpretations. Initial codes were generated to highlight key content and meaning. Codes were then clustered together via an inductive process. Theme comparison generated descriptive themes before further interpretation through discussions (Y.C., S.C., and E.J.M.), to initiate analytical themes, alongside a list of superordinate and subthemes. Analysis discussions were held to refine themes and support reflexivity. Y.C. returned to the original papers frequently to ensure analysis was appropriately contextualized.

### 2.5 Reflexivity

The research team brings significant clinical and research experience with people with ALS, from neurological and psychological approaches. As a team we align with acknowledging and prioritizing support for emotional experiences of HCPs working with this population. This direct clinical experience of the topic being studied allowed greater depth of discussion and interpretation. A reflexive journal, in which the authors recorded their thoughts and reflections throughout the data analysis, was kept. The reflexive journal helps to ensure greater awareness and discussion of the personal views and biases of the authors that could impact interpretation.

## 3 Results

### 3.1 Summary of Included Studies

**FIGURE 1:**
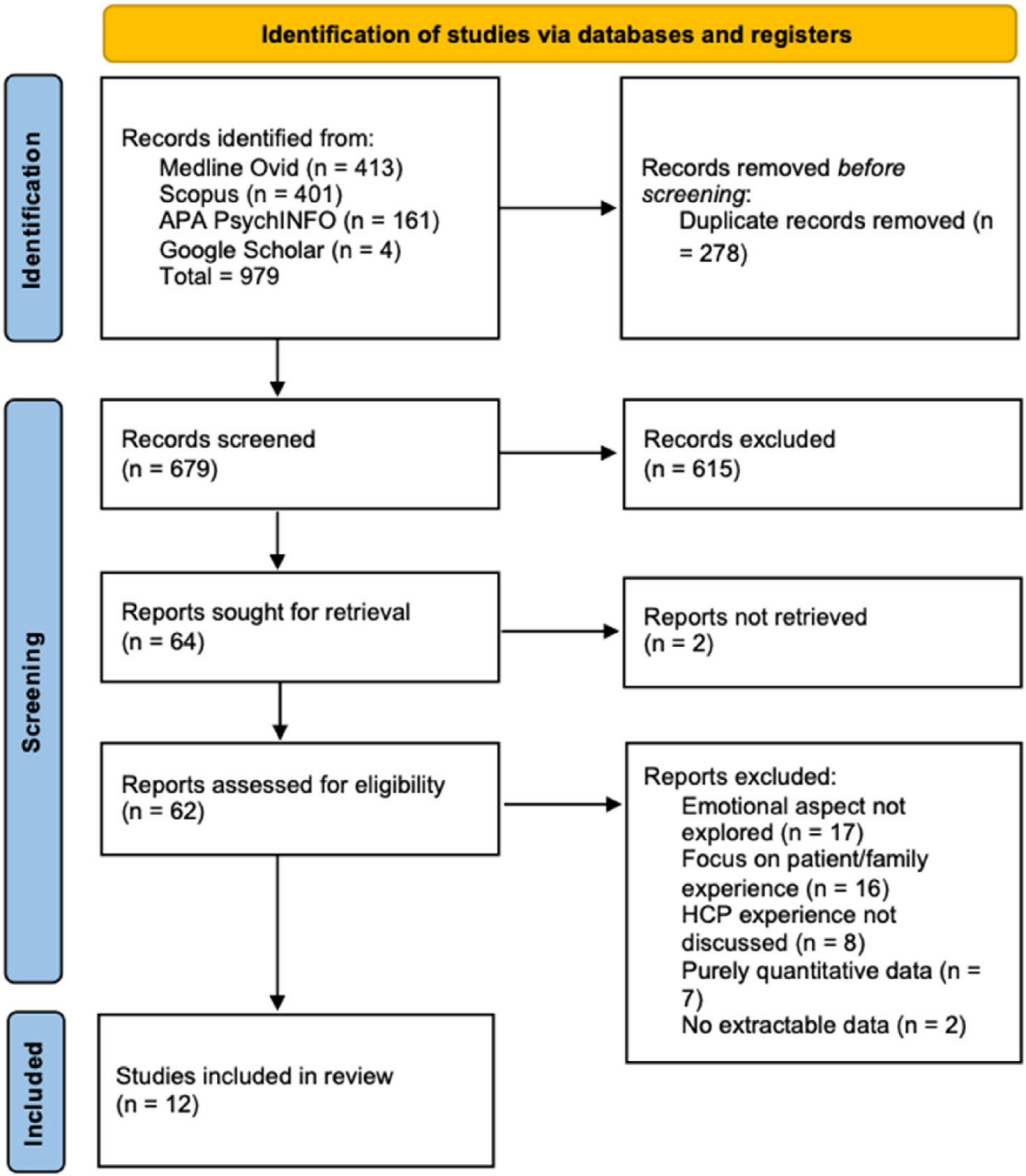
PRISMA flow diagram.

Searches across databases yielded 679 studies, and following de‐duplication 615 studies were eliminated following title and abstract screening, with 64 papers subject to full‐text review (see Figure 1). Twelve reports were included, consisting of nine original journal articles, two doctoral dissertations and one service evaluation report. Study characteristics are detailed in Table 1.

**TABLE 1:** Overview of study characteristics.

Author (year)	Country	Participant characteristics	Recruitment	Data collection and methodology	Key themes	Quality
*Peer‐reviewed publications*						
Anestis et al. [24]	UK	*N* = 19 nonmedical HCPs working with motor neurodegenerative conditions Aged 28–64, 17 females, 2 males 6–43 years' experience	NHS Trusts, snowball sampling and social media advertisements	Semistructured interviews Thematic analysis	Dealing with the diagnostic aftermath Unpacking the diagnosis Breaking bad news as a balancing act Empowering patients to regain control	High
Anestis et al. [25]	UK	*N* = 8 neurologists working with motor neurodegenerative conditions Aged 38–54, 6 males, 2 females 2–20 years' experience	NHS Trusts, and snowball sampling via social media advertisement	Semi‐structured interviews Interpretative phenomenological analysis	Meeting patients' emotional and information needs at diagnosis: a balancing act between disease, patient and organization‐related factors Empathy makes the job harder: the emotional impact and uncovered vulnerabilities associated with breaking bad news’	High
Beyermann et al. [26]	Sweden	*N* = 11 nurses working with people with ALS Aged 41–61, 10 females, 1 male 4–21 years' experience	Via distribution of an invitation and information letter to randomly selected home care units	Semi‐structured interviews Content analysis	To support in an increasingly difficult everyday life To support in emotionally challenging situations	High
Brown [27]	UK	*N* = 9 ALS MDT members 8 females, 1 male 1–> 100 case experience	Advertisement in local newspaper and local groups, MDT identified by one pair of participants	Semi‐structured interviews described as “conversations” Hermeneutic analysis	Strengths of professional role Ideals of care Role limitations Situated friendship Learning through experience to care	High
Cox et al. [28]	UK	*N* = 8 HCPs working with people with ALS	Email invitation to identified HCPs	Semi‐structured interviews Thematic analysis	Experiences of the withdrawal process The team Emotional impact APM (2015) guidance	High
Faull et al. [29]	UK	*N* = 130 doctors working with people with ALS	Email to members of the Association of Palliative Medicine of Great Britain and Ireland	Categorical and free text questionnaire Thematic analysis, grounded theory, and descriptive analysis	Practical challenges Ethical challenges Emotional challenges	High
Gamskjaer et al. [30]	Denmark	*N* = 12 HCPs working with people with ALS Aged 30–59, 12 female, 0 males <5 –> 10 years' experience	Invitation to all HCPs within 2 MND teams	Focus groups Structural analysis and critical analysis	Fundamental drive Working conditions Value of collegiality Work life balance	High
Hansen et al. [31]	Canada	*N* = 85 HCPS working with people with ALS 59 females, 29 males age > 20–> 60 1–26 years in practice	Survey link sent to Canadian ALS Physician network via email, forwarded to other professionals snowball sampling	Free text questionnaire Thematic analysis	Challenges in ALS care provision Rewards in ALS care provision Institutional support for wellness	High
Olesen et al. [32]	Denmark	*N* = 9 HCPs working with people with ALS and cognitive impairment 8 females, 1 male 1–6+ years' experience	Via contact with key leaders within health and social care, who selected HCPs meeting inclusion criteria	Focus group, semistructured interviews Interpretive Description	Collaboration a balancing act Working in a home of sorrow Coordinating threads to tie	High
*Service evaluation*						
Hunt et al. [33]	UK	*N* = 7 HCP working in neurorehabilitation of patients with conditions including ALS 3–20 years' experience	Purposive sampling within one neurorehabilitation service	Semi‐structured interview Thematic analysis	A race against time Dealing with death A double‐edged sword	High
*Thesis*						
Dilley [34]	UK	*N* = 6 doctors specializing in ALS 2+ years' experience	Direct contact to participants via email	Semi‐structured interviews Interpretative phenomenological analysis	‘It's enjoyable, it's depressing, it's important’ The boundaries between self and other 3. ‘People die’	High
Slay [35]	UK	*N* = 10 HCP working with people with ALS 8 female, 2 male 6 to 20+ years' experience	Purposive sampling through contacts in health care trusts and services via email	Semi‐structured interviews Interpretative phenomenological analysis	Death is part of the job Nuanced challenges in MND Places death on your personal agenda Caring for oneself to care for others	High

Abbreviations: ALS: amyotrophic lateral sclerosis, HCP: healthcare professional, MDT: multidisciplinary team, MND: motor neuron disease, NHS: national health service, UK: United Kingdom.

Papers were published from 2003 to 2024, in the United Kingdom (*n* = 8), Denmark (*n* = 2), Sweden (*n* = 1), and Canada (*n* = 1). Studies collected data via semi‐structured interviews (*n* = 8), focus groups (*n* = 1), which one paper described as “conversation” with primary and secondary questions [27], focus group plus individual interviews (*n* = 1) and mixed method questionnaires (*n* = 2). Where papers examined the perspectives of multiple groups of stakeholders; only data relating to HCPs was extracted. Nine studies included data from HCPs within ALS exclusively, two studies [24, 25] focused on HCP experiences in a range of neurodegenerative diseases; quotes were only used when it was clear that the quote related to ALS, either because it was explicitly stated or because the participant's role was ALS specific. One service evaluation [33] included HCPs working in neurorehabilitation, but the service evaluation was ALS specific. All included studies were found to be of high quality using the CASP checklist.

### 3.2 Data Synthesis

**FIGURE 2:**
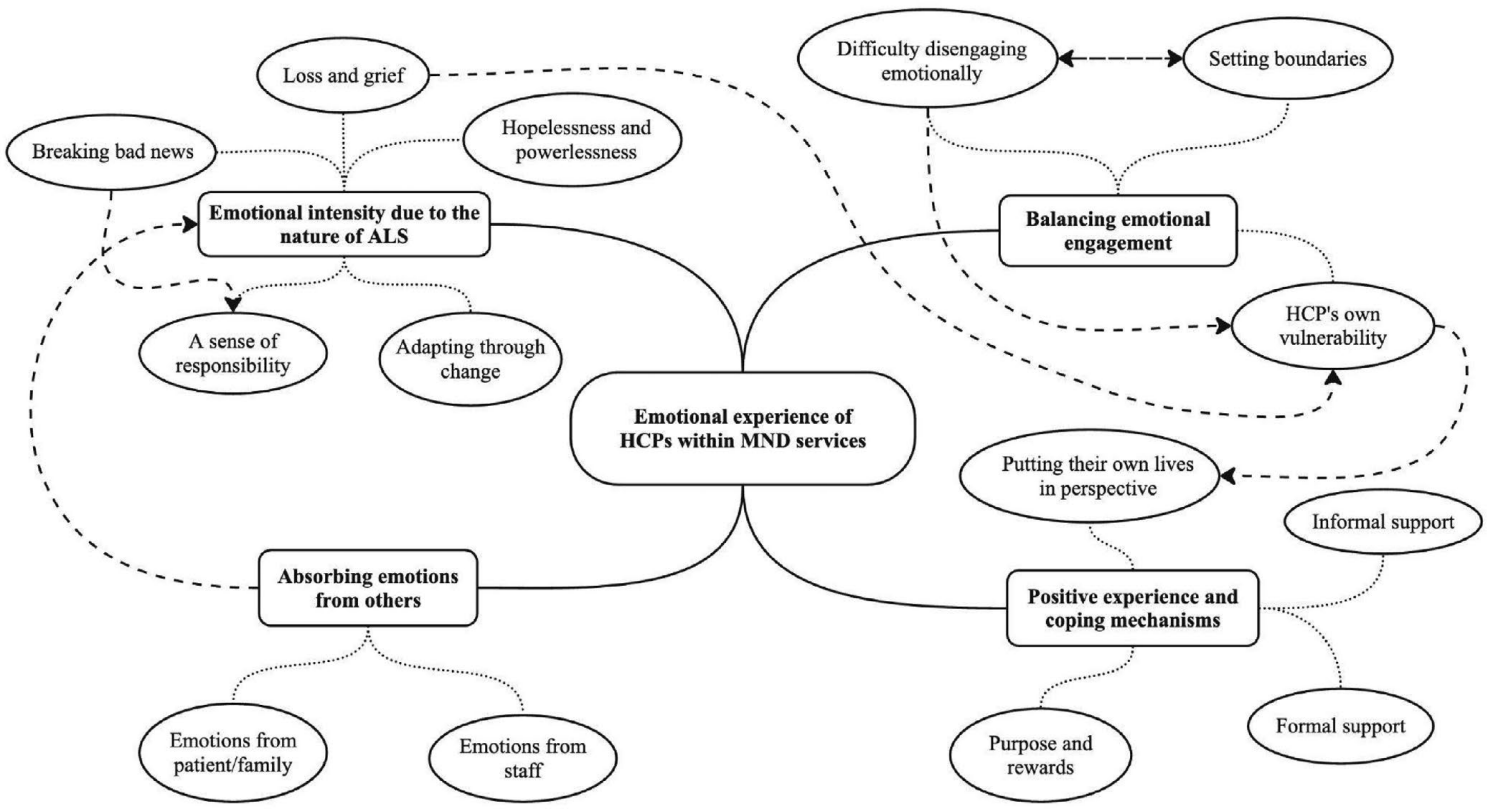
Overview of the inter‐relationship of the main themes and subordinate themes. Solid lines represent main themes, dotted lines represent subordinate themes from each main theme, and dashed lines with arrows represent the inter‐relationship of themes contributing to each other.

Four main themes were developed: (1) emotional intensity due to the nature of ALS, (2) absorbing emotion from others, (3) balancing emotional engagement, and (4) positive experiences and coping mechanisms. The main themes were further divided into 14 subordinate themes, with links between them noted, as summarized in Figure 2.

#### 3.2.1 Theme 1: Emotional Intensity due to the Nature of ALS

HCPs experience specific emotional challenges while working with people with ALS. Managing ALS on a day‐to‐day basis is challenging, and while the emotional responses described are not unique to ALS, they are often more intensely felt due to its progressive and terminal nature.

##### 3.2.1.1 Breaking Bad News

The emotional weight of managing ALS affects HCPs throughout the patient journey. Although an essential part of clinicians' roles, the diagnosis of ALS is particularly emotionally taxing due to HCPs' knowledge regarding the progression of the disease.

… all participants agreed that BBN [breaking bad news] was emotionally difficult, an unavoidable but significant part of their role. During and after BBN, HCPs reported sometimes feeling “drained” (P5, P19), “exhausted” (P5), “sad” (P1, P7, P8) and “anxious” (P8) and had sympathy for the patients, especially knowing how these conditions would progress and that they would have to give more bad news as the condition deteriorated. [24]

HCPs question themselves after delivering the diagnosis and breaking the news of such a significant condition. HCPs find this emotionally draining and burdensome, and question their abilities and clinical skills.

…I always feel completely exhausted, and I've noticed my communication skills plummet after this [delivering the diagnosis]. I replay the consultation in my head and think ‘could I have done it better?’, ‘was that the right thing?’ [25]

Professionals felt limited competence and confidence in breaking bad news, such as delivering an ALS diagnosis. These feelings caused a knock‐on effect, negatively impacting subsequent clinical conversations, interactions with colleagues, and/or personal relationships outside of work.

##### 3.2.1.2 Losses and Grief

ALS causes frequent losses, including loss of function, loss of independence, and changes in identity and relationships for people living with ALS and their families. HCPs witness and sometimes identify with this grief, and describe experiencing their own grief when people they care for die.

The investment and relationships built with these patients leads to a professional grief, acknowledged to be different for each individual. [28]

Participants saw their responsibility being to hold the emotional impact of ALS for the person diagnosed and their family around them. This meant they could not always find space for their own grief.

[There is an] emotional impact of creating a space for patients and their families to grieve the illness, their current loses and future loses, walking with patients through their journey with ALS when it is very difficult for patients to find moments of hope/joy in the face of this illness. [31]

##### 3.2.1.3 Hopelessness and Powerlessness

HCPs also struggle with the feeling of hopelessness and powerlessness relating to the incurable nature of ALS and the lack of options to alter disease progression. They reflected on a lack of management options to alter disease progression.

Being exposed to patients' intense emotional reactions was experienced as an additional source of distress for professionals, eliciting sadness and sympathy for patients but also a feeling of powerlessness… ‘I think it's feeling powerless. People say that knowledge is power, but with incurable conditions, it doesn't feel like that. So, I'm dragging them into giving them bad news and I can't really make’ [25]

Participants felt personally responsible for carrying this burden.

We get so affected by it [ALS] because it is so hopeless, and the hopelessness…it becomes such a heavy burden to carry. [32]

Despite HCPs' efforts to offer solutions and meaningful help, the incurable nature of ALS and its relentless progression mean that deterioration and death are inevitable.

[There's a] feeling of inability to truly help these patients. We know the outcome of the disease and even though we try to help, it feels sort of inconsequential. [31]

Participants felt a sense of frustration, defeat and low control over the situation.

They know it's coming, you know it's coming, and I think it's the progression of the condition along the way that makes it for me harder than working with other people – so that day‐to‐day‐change in ability to function. [33]

##### 3.2.1.4 A Sense of Responsibility

A sense of responsibility, even guilt at times, was felt. Participants were aware of the life‐changing impact of their words and the importance of sharing information sensitively.

patients “will never forget the way they were told the news”. [24]

Participants expressed feeling almost at fault for the misfortunes faced by people with ALS. This feeling of responsibility is most apparent when HCPs are performing an action which they perceive to be detrimental to the person's wellbeing, such as delivering the diagnosis or withdrawing ventilation within end‐of‐life care.

There's always a moment, just before you say the name of the disease, where you feel terribly responsible. Like you've done it to them, that you've given it to them, I don't know if it's just me. I don't know why it feels like in diagnosing that you own the disease, or sort of passing it through to them. That's very sad. [25]

HCPs acknowledged the contradiction in feeling that they are causing harm or distress while doing their job, but struggle to stay unaffected by the emotional consequences. This was particularly relevant when aligning with the person's wishes around their end‐of‐life care.

Perhaps the most complex emotional issue was related to death being so related to an action [withdrawal of ventilation]. “It feels like I am causing their death even though I am carrying out their wishes and it is in fact a horrible disease that is killing them. My rational brain can eventually navigate the arguments but my heart lags a long way behind.” [32]

##### 3.2.1.5 Adapting Through Change

The heterogeneity in clinical presentation and individual preferences and values, alongside the rapid progression and change in patient needs in ALS, presents many challenges that HCPs must balance and manage.

Professionals reported adjusting their demeanour and broadening their value perspectives to meet those of the patient; trying to fit in with their lifestyles and stance; using emotional effort to be responsive to patient needs and keeping the focus on the patient. [27]

HCPs must adapt constantly, both within and between each patient and their family. While such emotional maneuver is necessary to provide high quality care, it can add to HCPs' emotional burden and fatigue.

Moving from one patient to another becomes quite a challenge. You may be having to be very serious and very sad and perhaps going to someone who's completely opposite and it's hard then to quickly change’. [27]

#### 3.2.2 Theme 2: Absorbing Emotions From Others

HCPs often find themselves absorbing emotional responses from others, in addition to managing their own emotions, which becomes a further source of distress.

##### 3.2.2.1 Emotions From Patients/Family

The loss of independence in daily living can become extremely burdensome and stressful to both patients and their family carers. This is increasingly made difficult as the disease progresses, sometimes at a rate so rapid that people with ALS and their family struggle to adapt to and process emotionally. HCPs can therefore be on the receiving end of such negative emotions; this can be distressing and burdensome.

… I tend to be there to soak it all up ‘cos sometimes they need to offload and you've actually got to take on their burden of grief. [27]

Sometimes frustration and aggression with the condition are directed toward HCPs by people with ALS and their families.

This led to fear of home visits, worrying about the emotional reactions HCPs would be faced with

Because of the mood swings of the family members, they could sometimes experience discomfort during the home visit and even fear. [26]

##### 3.2.2.2 Emotions From Staff

The emotional burden for HCPs is felt mostly on a personal level; however, there are times when a common emotional response is felt across a team. An example of this is the withdrawal of noninvasive ventilation (NIV), a decision made by some people living with ALS at the end of their life.

I found dealing with the (very highly expressed) emotions of staff watching, assisting or aware of the withdrawal of NIV in a patient was more challenging for me than the emotions of the family. [29]

HCPs with a leadership role supporting such decisions therefore feel the burden of managing distress among staff and their ethical concerns.

The emotional burden which was felt in terms of managing the emotions of others (patient, family and staff) throughout the process was the commonest theme to emerge. Supporting others and conflict resolution formed part of this burden. [29]

#### 3.2.3 Theme 3: Balancing Emotional Engagement

Over time, participants' emotions become intertwined with their experiences caring for people with ALS, influencing their own behavior in subtle, often involuntary ways. To cope, many HCPs adopt specific emotional management strategies.

##### 3.2.3.1 Difficulty Disengaging Emotionally

The intense emotional experience of working in ALS leads HCPs to become emotionally invested, and some find it difficult to disengage from this.

The feeling of having been out there listening to how difficult it is for the people with ALS and talk about death and how they're going to die. Sometimes it's just a bit difficult to come home and sit down and have a nice family dinner. [30]

Participants reflected on the impact of struggling to disengage from work.

Others explained how they often ended up engaging themselves a little extra because they became so affected by the severity of the situation that it made it hard for them to leave their jobs behind when they were not working [32].

The contrast in disease severity seen in ALS compared to other neurological conditions reduced empathy with people with other diseases.

As the HCPs developed more experience with the client group there was an increased risk of compassion fatigue. This was exacerbated by the severity of {ALS} compared with the presentation of other neurological conditions: ‘…you've got this person with {ALS}, and then you go to someone else's house and they might have mild Parkinson's, but sat there telling you how dreadful their life is. [33]

Another participant described their early experiences with ALS as *“haunting”* and felt they had experienced symptoms consistent with post‐traumatic disorder, exemplifying how psychologically damaging it can be for HCPs to work in this sector without effectively understanding and regulating their own emotions.

Certainly, I think some of the things that I felt early on … would certainly mimic PTSD. Anxiety, hypervigilance … flashbacks—that sort of thing, because it's just haunting. [33]

##### 3.2.3.2 Setting Boundaries

Some participants dissociate themselves from their work‐related emotional load. This gives them the space to manage their own emotions without getting personally involved or hindered by the volume and intensity of emotions.

I have to stay there (after diagnosis disclosure) and probably manage my emotions by trying to feel a bit numb. So, using a kind of—“I'm watching myself giving bad news“ rather than “I'm feeling myself giving bad news”. [25]

For some participants, this boundary is set purposefully in virtue of professionalism, seen as a necessity to maintain a healthy clinician–patient relationship.

Firstly, as echoed by all participants, was the importance of preserving emotional boundaries and distance, and how part “of being a professional is to be slightly out of the room but in the room.” [34]

For some, an overflow of personal feelings in patient encounters would be considered unprofessional. Becoming overly involved emotionally is frowned upon and counterproductive.

Conversely, some HCPs see experiencing the emotional impact as important and feel it should be normalized. The empathetic response was felt to be essential to providing good care for people living with ALS.

I can't see myself doing the job well if I had no sense of the sadness at someone with an unexpected diagnosis of a life‐shortening condition that's going to disable them and cause their premature death. I think if you don't get that that's sad then stop and re‐group. [35]

Setting boundaries for the level of emotional engagement is a balancing act of fine margins.

… We're not robots clearly and we have to do, I like to think I do, have…that empathic approach um but at the same time I don't think you'd be a good doctor … if that became too prominent…it's about that professionalism and you're there to provide information to the patients, try to give them any management therapies that might be available. [35]

##### 3.2.3.3 HCPs' Own Vulnerability

As HCPs experience the patient journey alongside people living with ALS and informal carers, they draw parallels between the patients' lives and their own, which prompts a sense of vulnerability.

Delivering a diagnosis to patients with a similar age, gender or family circumstances to them was more upsetting for some neurologists. Breaking bad news in these cases ‘brought it home’ (P3) and acted as a reminder of participants' own vulnerability and their own fears regarding the unpredictability of life [25].

Additionally, sometimes HCPs have less capacity to cope with distress associated with ALS due to stress or vulnerability in their own lives.

Participants described how their individual situation affected how susceptible they were, for instance during fragile periods of life, where they tended to get more affected by the presence of illness and death compared to periods of energy. [30]

For some participants, the effects of working with people living with ALS extend beyond psychological. One participant developed benign fasciculation syndrome (BFS) when first working in ALS.

members of the team had suffered from benign fasciculation syndrome (BFS)—a rare benign disease that mimics some of the symptoms present in {ALS}. The onset of BFS appeared to occur when the HCPs were relatively inexperienced: ‘…when I first started working with people with ALS, I would say within the first six months, I started to get fasciculations.’ [33]

Another participant alluded to associating such symptoms with ALS, despite having the knowledge that fasciculation is a common, benign finding in many.

Anyone working in this sort of field notices things on their own body … one of the signs in {ALS} are fasciculations, which are involuntary muscle twitches … but they're also very common in the general population … So I get them, my boss gets them and when you're working in the field…you can rationalise it … because you know they're actually normal and there are no other features but {ALS} is a very rare disease. [35]

#### 3.2.4 Theme 4: Positive Experiences and Coping Mechanisms

Positive emotional experiences keep HCPs devoted to their roles in ALS care, and they use multiple strategies to cope with the emotional burden of their roles.

##### 3.2.4.1 Putting Their Own Lives in Perspective

Witnessing the progression of disease in people with ALS increases some HCPs' realization of the uncertainty and fragility of life.

being cognisant that {ALS} is a progressive disease with a limited prognosis for the patients they support also provided the HCPs with an impetus to live life to the full: ‘I get on with my life … I want to do everything I can do, I want to go on holidays every year. So, my outlook is wider … than what it used to be ten years ago—before I started this job.’ [33]

While this sense of vulnerability can be distressing, some participants use this to bring a positive change in their outlook on life; they learn to appreciate and enjoy life more.

I've watched several people, they're planning their lives and then suddenly they've got motor neurone disease and it's rapidly progressive and they're dead within a year um you know, making you think … you've got to be happy with life right now, you can't always be thinking ahh I'll be happy with life in time … You've got to be sure that you're doing the right things now and that if something devastating happened tomorrow that you haven't just put off stuff. [35]

Some participants took a reflective approach, comparing theirs and their patients' lives directly.

Many participants reported increased use of comparative coping by contrasting aspects of their lives to their patients to generate a greater sense of appreciation, as participant 6 explained: “Oh, this isn't so bad, just think about what my patients go through and that's actually quite a positive thing to be honest because you just think, well you can get through this don't be so anxious, far worse things can happen.” [34]

##### 3.2.4.2 Purpose and Rewards

Despite the challenges, participants described working with people with ALS as a privilege and rewarding.

Although staff may not be able to alter the disease course, they believe their work dignifies the experience of ALS, which empowers them and provides a sense of purpose.

Sharing deeply private and emotional conversations with patients and families, knowing that someone trusts you enough to open their hearts [is rewarding]. I'm privileged to have the chance to share these most intimate moments with patients and families. [31]

Ensuring the dignity of people with ALS at the end of their life was important.

(I felt) really proud, it was an incredibly dignified thing to do for someone and it might be one of the things I've been most proud of in my career. I felt proud to have been part of giving someone a way out of pretty much the worst thing in the world. [28]

##### 3.2.4.3 Informal Support From Colleagues

While participants did not frequently describe coping strategies, most staff channeled their emotions through the support from their colleagues, often in the form of an informal chat between colleagues after a difficult encounter.

I think it's really important that you have someone to lean on. That you're confident and trust, that if I suddenly think something is tremendously difficult, well, then I can lean on some of my colleagues. [30]

When experiencing emotional distress, HCPs look to each other, knowing that they understand each other's experiences and challenges with ALS.

I went to the car and cried and felt like I can't handle it … oh then I'm like this no I've calmed down, I am talking to a colleague, I had to call and vent … that this is not my fault, this is a frustration, this is chaos, but now I calm down and can talk to my colleague, I go back. [26]

##### 3.2.4.4 Formal Support

Many participants expressed the need for more formal, structured support infrastructure for HCPs working with people living with ALS, such as debriefing and team building and wellness exercises. Those who have had access to support systems reported finding it helpful, especially after an emotionally significant event.

The post‐withdrawal debrief, whether formal or informal, was cited by many as being helpful: “…the debrief helped, just to know that you guys had found it tough as well.”… They emphasized the importance of being offered a 1:1 debrief as they would not have felt comfortable airing their frustrations in front of the team [28].

However, this area of staff wellbeing management is lacking and could play a pivotal role in mitigating emotional challenges.

Given the challenging interactions and emotionally charged conversations inherent in ALS care, the majority participants felt they would benefit from more consistent team building exercises and debriefing. They also felt that formal training to deal with grief and burnout would be valuable. Provision of counselling and participation in wellness exercises were encouraged. [31]

Opportunities to access support from psychologists were limited. Where this was available participants reported positive experiences, being guided through their emotions in reflective discussions.

There was recognition of the benefits of reflecting with colleagues “who really understand and are going through the same thing” (P8) within a space where it feels safe to disclose your honest experiences, as participant 9 explains: “We have a really good psychology team that we work with, that are very aware that a lot of the patients that I work with day to day are emotive for all staff members” [34].

## 4 Discussion

This systematic review highlights the intense emotional experiences of working in ALS, building on a recent scoping review [19]. Some HCPs experience significant psychological and physical effects associated with their role [24, 27, 29, 30, 33, 34]. HCPs witness and experience distress related to progression and the death of those for whom they care, while taking on the distress of others [26, 27, 29]. Given the nature of ALS, these emotional experiences are not only significant but recurring, leaving HCPs to grieve and process distress while moving from one patient encounter to the next, adapting to each person's needs, building new relationships, and acknowledging future losses and grief. HCPs respond to these emotions in different ways. Some distance themselves from their own emotions to provide high‐quality care [25, 27, 29], while others perceive the need to feel and stay close to the distress of ALS as integral to their role.

Many of the reported emotional experiences are associated with the devastating nature of ALS and feelings of powerlessness [25, 30, 31, 32, 33, 34] related to limited effective disease‐modifying therapies [1, 8]. This feeling of powerlessness affects HCPs in all roles, even when they are not responsible for delivering the diagnosis. Despite the challenges and distress associated with working in ALS, HCPs value their work and benefit from it, having a greater appreciation for their own lives [33, 34, 35] and feeling privileged to support people with ALS [28, 30, 31]. HCPs find a range of coping strategies useful for dealing with their work's emotional intensity [25, 26, 28, 30, 34].

Discussions of positive experiences and coping mechanisms were relatively scarce across included studies. Most participants relied on seeing the inherent, immense value in their work and patients' appreciation of this as motivation to continue [28, 30, 31, 34]. Informal peer support appears to be integral [26, 30, 31, 35], but only one study [28] reported using debriefing as a form of formal support, likely due to the lack of formal support systems available. Limited access to a psychologist was noted, although where this was present, the support was useful [24, 34]. Currently, formal psychological training and supervision is more readily available within other areas of medicine, such as palliative care [36, 37]. The lack of psychological support for staff in ALS is a gap that warrants attention.

A balancing act of emotional involvement exists in ALS care, with some clinicians leaning strongly toward professional distance as necessary [30, 32], and some leaning toward the importance of emotional engagement [35]. The emotional challenges described by HCPs are not unique to ALS, but the nature of ALS, the associated symptoms, and the lack of effective treatments contribute to the sense of powerlessness and the intensity of emotions experienced by staff working in ALS. Staff who work in other areas of medicine that share characteristics with ALS, such as palliative care and oncology, experience some similar challenges [38, 39, 40, 41]. A similar theme of seeking emotional balance was reported for HCPs dealing with life‐threatening illnesses [41]. This balance poses huge consequences for HCPs' emotional wellbeing and work–life balance, which influences job satisfaction, productivity, and patient outcomes [42, 43]. Issues such as burnout [31] and compassion fatigue [33] are frequently described [44] in the literature looking at staff experiences of other life‐threatening illnesses, but until recently had not been fully explored for staff working in ALS.

## 5 Clinical Implications

HCPs require additional support to continue to care effectively for people living with and affected by ALS. Some of the things HCPs reported finding useful included reflecting on the value of the work they do, the way the work shapes their own life choices, and accessing peer and formal support. The existing coping strategies HCPs have should be built upon, for example, by offering opportunities to reflect on their work, relate this to their own values as clinicians and people, debrief with other HCPs, and have access to formal structures for support, such as clinical psychology. This is important, given the unmet psychological needs and high prevalence of work‐related emotional difficulties among HCPs [18]. Training is needed to support HCPs to cope with the stress and distress of working in ALS, build healthier therapeutic relationships with those they care for, and experience improved wellbeing.

The principles of acceptance and commitment therapy (ACT) were alluded to by participants. Key skills related to ACT are acceptance (willingness to face emotional challenges) and mindfulness (noticing thoughts, emotions, and sensations in a nonjudgmental way). An adapted version of ACT was recently found to improve quality of life for people with ALS [15], which suggests this model is relevant in an ALS context. The quotes from professionals about deriving meaning and value from supporting people with ALS and their families align with the ACT model; by providing ACT training and supervision to HCPs, it would be possible to build on this existing coping strategy used by HCPs and develop additional skills.

## 6 Limitations

Our inclusion criteria included qualitative research only and publications written in English, meaning studies that were not translated into English were missed. Studies that briefly discussed HCPs' emotional experience without extractable data were excluded, even though they may have had important points to contribute. The review focused on HCPs working in ALS and did not specifically search for social care professionals such as social workers, who in some countries are not part of multidisciplinary ALS clinics. It also did not search for paid carers, who provide residential support to many people with ALS. There is likely to be overlap in emotional experiences between these professionals and the HCPs described in this review, and their needs must also be considered.

## 7 Conclusion

HCPs working in ALS experience multi‐faceted emotional challenges, with few coping mechanisms and support systems in place to process them effectively, and this may have implications that ultimately affect patient care. There is an unmet demand for formal support, such as debriefing, supervision, and further training on how to deal with distressing emotions. This should ideally be facilitated by staff experienced in both ALS and psychology. Further research to develop and explore the effectiveness of psychological interventions for ALS HCPs is needed.

## Author Contributions

**Esther V. Hobson:** writing – review and editing, supervision, funding acquisition. **Emily J. Mayberry:** conceptualization, funding acquisition, writing – review and editing, project administration, supervision, investigation. **Jess Mere:** investigation, writing – original draft, data curation, writing – review and editing. **Christopher J. McDermott:** writing – review and editing, supervision, funding acquisition. **Charlotte Wright:** writing – review and editing, supervision. **Alys Wyn Griffiths:** investigation, methodology, writing – review and editing, supervision, funding acquisition. **Sarah Creer:** investigation, methodology, writing – review and editing, formal analysis, supervision. **Young Chan:** investigation, writing – original draft, writing – review and editing, formal analysis, data curation.

## Funding

This work was supported by the Motor Neurone Disease Association (943‐794).

## Ethics Statement

We confirm that we have read the Journal's position on issues involved in ethical publication and affirm that this report is consistent with those guidelines.

## Conflicts of Interest

The authors declare no conflicts of interest.

## Supporting information


**Data S1:** mus70309‐sup‐0001‐Supinfo1.docx.

**Data S2:** mus70309‐sup‐0002‐Supinfo2.docx.

## Data Availability

The data that support the findings of this study are available from the corresponding author upon reasonable request.
