# Supplementary material for: The Emotional Experiences of Healthcare Professionals Working in Amyotrophic Lateral Sclerosis: A Systematic Review
Source: Muscle Nerve. 2026 Jun 18;74(3):517–29. doi: 10.1002/mus.70309 (PMC13471968; doi:10.1002/mus.70309)
Supplement: Supplementary file 1 [file MUS-74-517-s001.docx]

Eligibility criteria.

Inclusion criteria

Samples include HCP participants who work with people living with ALS

Studies that explore the emotional experiences of HCPs working with people with ALS

Studies employed qualitative methodology and analysis, including mixed-method studies

Studies are written in the English language

Exclusion criteria

Studies which utilised solely quantitative design

Studies that do not have direct quotations from HCPs about their emotional experiences of working with people living with ALS

ALS: amyotrophic lateral sclerosis, HCPs: healthcare professionals.
