# Supplementary material for: The Emotional Experiences of Healthcare Professionals Working in Amyotrophic Lateral Sclerosis: A Systematic Review
Source: Muscle Nerve. 2026 Jun 18;74(3):517–29. doi: 10.1002/mus.70309 (PMC13471968; doi:10.1002/mus.70309)
Supplement: Supplementary file 2 [file MUS-74-517-s002.docx]

What are the emotional experiences of healthcare professionals working with people living with motor neuron disease (MND)?

Medline Ovid = 413

APA PsycINFO = 161

Scopus = 401

Total = 975

Total after duplicates removed = 698

Search strategies

Ovid MEDLINE(R) Epub Ahead of Print and In-Process, In-Data-Review & Other Non-Indexed Citations and Daily <December 10, 2024>

<https://ovidsp.ovid.com/ovidweb.cgi?T=JS&NEWS=N&PAGE=main&SHAREDSEARCHID=6UyOItlbAejBodNvwuoNv2ojSKYUTQ5FN8UDf7xx1xBC967rUyoRZrA64PygcM9xm>

1	exp Motor Neuron Disease/

2	(motor neuron* disease* or mnd or amyotrophic lateral sclerosis).mp.

3	1 or 2

4	exp Health Personnel/ or exp Professional-Patient Relations/

5	exp Attitude of Health Personnel/ or Burnout, Professional/ or exp Emotions/ or exp Stress, Psychological/ or exp Occupational Stress/ or exp Anxiety/ or exp Anger/ or exp Guilt/

6	4 and 5

7	((staff or professional* or worker* or medic? or physician* or doctor* or clinician* or therapist* or dentist* or psychiatrist* or surgeon* or nurse* or psychologist*) adj6 (emot* or distress or stress or burnout or moral injury or empathy or experience* or attitude* or perspective* or perception* or view* or psycholog* or challenge* or well being or wellbeing)).mp.

8	6 or 7

9	3 and 8

10	limit 9 to english language

APA PsycInfo <1806 to December 2024 Week 1>

<https://ovidsp.ovid.com/ovidweb.cgi?T=JS&NEWS=N&PAGE=main&SHAREDSEARCHID=5EehkjUk2TGx22nk1x3TF7D6DjYRDI8lcxCQ7DTC771KM14KMvHeINOKpLqVoLhGU>

1	(motor neuron* disease* or mnd or amyotrophic lateral sclerosis).mp.

2	exp Health Personnel/

3	exp Health Personnel Attitudes/ or exp Job Burnout/ or exp Emotions/ or exp Occupational Stress/ or exp Stress/ or exp Distress/

4	2 and 3

5	((staff or professional* or worker* or medic? or physician* or doctor* or clinician* or therapist* or dentist* or psychiatrist* or surgeon* or nurse* or psychologist*) adj6 (emot* or distress or stress or burnout or moral injury or empathy or experience* or attitude* or perspective* or perception* or view* or psycholog* or challenge* or well being or wellbeing)).mp.

6	4 or 5

7	1 and 6

8	limit 7 to english language

Scopus

( TITLE-ABS-KEY ( ( "staff" OR "professional*" OR "worker*" OR "medic?" OR "physician*" OR "doctor*" OR "clinician*" OR "therapist*" OR "dentist*" OR "psychiatrist*" OR "surgeon*" OR "nurse*" OR "psychologist*" ) W/6 ( "emot*" OR "distress" OR "stress" OR "burnout" OR "moral injury" OR "empathy" OR "experience*" OR "attitude*" OR "perspective*" OR "perception*" OR "view*" OR "psycholog*" OR "challenge*" OR "well being" OR "wellbeing" ) ) AND TITLE-ABS-KEY ( "motor neuron* disease*" OR mnd OR "amyotrophic lateral sclerosis" ) ) AND ( LIMIT-TO ( LANGUAGE , "English" ) )
